# Determine the optimal ignition timing function based on combustion duration, load and fuel blending ratio of an engine powered with diesel-methanol blends

**DOI:** 10.1371/journal.pone.0351949

**Published:** 2026-06-22

**Authors:** Thong Duc Hong, Dinh Quang Phan, Son Hoang Do, Long Hoang Duong, Minh Quang Pham, Quan Thien Phan Nghiem

**Affiliations:** 1 Faculty of Transportation Engineering, Ho Chi Minh City University of Technology (HCMUT), Dien Hong Ward, Ho Chi Minh City, Vietnam; 2 Vietnam National University Ho Chi Minh City (VNU-HCM), Linh Xuan Ward, Ho Chi Minh City, Vietnam; 3 Faculty of Engineering & Technology, Nguyen Tat Thanh University, Ho Chi Minh City, Vietnam; GH Raisoni College of Engineering and Management Pune, INDIA

## Abstract

The study employs AVL Boost software to investigate the effects of different methanol blending ratios (BR) in diesel–methanol mixtures (ranging from 0 to 50%), ignition timings (IT), and combustion durations (CD) on the power and brake specific fuel consumption (BSFC) of a single-cylinder diesel engine under various loads of 85, 70, and 50%. Throughout this study, the term IT refers to the start of combustion of diesel fuel in the combustion chamber. The simulation model is validated by comparing the power and torque simulated results and experimental data, with the discrepancy less than 5%. The optimal ITs for maximum power and minimum BSFC are identified, then predictive models are developed based on these optimal IT values as functions of BR at various engine loads and CDs. The results show that the correlations between the IT, BR, CD, and engine load are linear functions. The ITs that simultaneously deliver peak power output and the lowest BSFC are governed primarily by CD, with the BR exerting a secondary effect; whereas, engine load plays a comparatively minor role. For maximum power, the largest variations in optimal ITs are approximately 15^o^CA for CD, 5^o^CA for BR, and 4^o^CA for load. For minimum BSFC, the corresponding maximum differences are about 15, 5, and 3^o^CA for CD, BR, and engine load, respectively. Optimal IT prediction models have been established that can minimize the time and cost associated with engine experimental tests to obtain the lowest BSFC and highest engine power at various CDs, engine loads, and methanol BRs.

## Introduction

Recently, governments around the world have introduced policies to control pollution from road vehicles. Among air pollutants, emissions from road motor vehicles account for a significant proportion. Further to the undeniable advantages, the operation of the internal combustion engine also has harmful effects on human health and the environment. Additionally, finding clean renewable energy sources is becoming increasingly popular due to the growing need for energy, rising oil prices, depletion of oil supplies, environmental degradation, and global warming related to the use of petroleum-based fuels [1]. Renewable biofuels have emerged as a promising solution for reducing pollutant emissions, thereby mitigating the increasingly severe greenhouse effect. This improvement can be explained by the ability of biodiesel to support cleaner and more efficient engine operation [2]. By reducing pollutant emissions and improving fuel utilization, optimized biodiesel blends play an important role in both boosting engine performance and promoting environmental protection [3]. Furthermore, biodiesel can be derived from a wide range of abundant feedstocks, such as water hyacinth (Eichhornia crassipes) [4] and chicken waste [5] … Alongside biodiesel development, the use of biodiesel blended with conventional fossil fuels, as well as the integration of emission reduction technologies, has become increasingly widespread. For instance, Kakran et al. investigated diesel–hydrogen–tri-ethylene glycol monomethyl ether [6] and diesel–hydrogen [7] in combination with exhaust gas recirculation and urea-based selective catalytic reduction to enhance engine performance while reducing emissions. Notably, the development and utilization of biofuels contribute to enhancing energy security by decreasing reliance on the importation and production of fossil fuels. Due to their oxygenation and regenerative nature, alcohol is becoming increasingly popular as a fuel for diesel-powered machinery. Due to its liquid state, excessive oxygen content, low cetane number, and potential for production from renewable resources, methanol has emerged as one of the most competitive alternatives to petroleum. Another point is that methanol production helps reduce CO_2_ emissions by using renewable biomass sources such as wood, sawdust, agricultural residues (rice straw, rice husk), corn, cassava, and woody crops. These feedstocks are gasified to generate synthesis gas (syngas, composed of CO and H_2_), which is subsequently converted into methanol [8–10]. The blending of methanol with diesel or gasoline has been extensively investigated for widespread application due to its advantages in emissions reduction, performance characteristics, and compatibility with existing internal combustion engines with minimal or no modification, thereby lowering the cost of transitioning to renewable fuels.

A stable diesel-methanol combination was achieved, and comprehensive investigations were undertaken to evaluate its performance and emission characteristics in a compression ignition engine. The use of biodiesel in diesel engines has been the subject of several investigations [11–14]. For example, prior studies have investigated the impact of injection pressure [11], the effects of varying mixture ratios [12], and the influence of pilot fuel injection [14] on methanol–diesel fueling strategies in diesel engines. In addition, several works have examined the application of ternary fuel blends, such as diesel/methanol/n-butanol mixtures, in diesel engine operation [13]. However, having a low energy level indicates that, to provide the engine with the same amount of energy, approximately double the amount of methanol as diesel fuel would have to be used. Due to its low cetane number and high latent heat of vaporization, methanol increases ignition delay. Furthermore, because methanol has less viscosity than diesel fuel, mixing, injecting, and atomizing it with air is simple [11,15]. And finally, the methanol-diesel mixture alters the fuel’s physical properties compared to pure diesel. Hence, to address these disadvantages, the engine needs to be modified in some specifications to suit the new fuel, achieving optimal effectiveness and efficiency. It was shown that the timing of injections has a major impact on almost every engine feature, as it affects how well the air-fuel mixture mixes, which, in turn, affects the combustion process as a whole.

Numerous experiments have been conducted to find out the optimal injection timings of the diesel engine fueled with biofuel. Hong et al. [16] identified the combustion phasing angle that enables a diesel-fueled engine to attain both peak power output and the lowest brake-specific fuel consumption (BSFC). The study identified the optimal angles through simulation and developed a predictive model for the optimal combustion angle corresponding to maximum power and minimum BSFC. Khatri et al. [17] investigated the best timing for fuel injection into a direct-injection diesel engine operating on a boiling combination of Karanj and diesel. Analysis of the experimental results indicates that the optimal injection timing for the Karanj–diesel blend occurs at 19 crank angle degrees (^o^CA) before top dead center (BTDC). Ganapathy et al. [18] used jatropha-derived biodiesel to examine the effects of injection timings on the compression ignition (CI) engine. The results from the experiment show that the least quantity of NO emission was achieved at an optimal injection time of 350^o^CA. An ignition timing (IT) of 340^o^CA corresponds to the condition at which the engine simultaneously produces minimum smoke opacity, unburned hydrocarbons, carbon monoxide, and BSFC, while achieving peak in-cylinder pressure, the highest heat release rate, and maximum brake thermal efficiency. Bhaskor J. Bora et al. [19] examined the optimization of injection timing in a dual-fuel diesel engine operating with raw biogas. At a compression ratio of 18, an advanced pilot injection timing of 29^o^CA BTDC was identified as optimal, producing the maximum brake thermal efficiency of 25.44% and a diesel substitution ratio of 82.67%, while also reducing CO and HC emissions to their minimum values. Nagesh et al. [20] analyzed the influence of injection timing on the performance of a diesel engine fueled with acid oil methyl ester. Their results demonstrated that advancing the injection timing to 27^o^CA BTDC is optimal for this fuel, as it delivers the highest brake thermal efficiency while simultaneously minimizing NOx, CO, and HC emissions. Banapurmath et al. [21] evaluated the performance of a small-bore direct-injection diesel engine fueled with Honge Oil Methyl Ester by employing three injection timings of 19, 23, and 27^o^CA along with varied injection pressures. The ideal injection time for Honge Oil Methyl Ester, according to their findings, is 19^o^CA BTDC for both performance and emissions. Sudarmanta et al. [15] examined the optimization of injection timing and injection pressure in a direct-injection (DI) 20C biodiesel engine prototype to enhance engine performance. The study identified 16^o^CA BTDC and 230 kg/cm² as the optimal injection timing and pressure, respectively, resulting in a 3.9% increase in power output and a 13.9% reduction in smoke emissions under high-load conditions. Nguyen et al. [22] determined the optimal combustion angles to attain the greatest power and lowest BSFC using AVL Boost software, based on different premixed ratios and combustion phases of diesel fuel, and subsequently developed a predictive model for the optimal combustion angle of diesel engines.

In particular, numerous studies have focused on identifying the optimal injection timing for diesel–methanol fuel blends in internal combustion engines in order to achieve improved performance and emission characteristics. Sayin et al. [23] conducted experiments using diesel–methanol blends in a diesel engine to investigate the effects of injection timing on pollutant emissions under different engine loads at a constant speed of 2200 rpm. Their results indicated that retarded injection timing (15^o^CA BTDC) led to reductions in NOx and CO₂ emissions, whereas advanced injection timing (25^o^CA BTDC) resulted in improvements in smoke opacity as well as reductions in unburned hydrocarbons and CO emissions. Li et al. [24] reported that, for a spark-ignition engine operating on methanol, advancing the injection timing to 39^o^CA BTDC together with an IT of 18^o^CA BTDC achieves the best trade-off between brake thermal efficiency and exhaust emissions. Sayin et al. [25] examined how variations in injection timing influence the performance and emission behavior of a diesel engine operating under methanol–diesel dual-fuel conditions. The findings indicated that an injection timing of 20^o^CA BTDC resulted in the most favorable BSFC and brake thermal efficiency values, while further advancement of combustion timing led to reductions in smoke opacity as well as CO and THC emissions. The effects of a methanol direct injection strategy on combustion behaviors and exhaust emissions were investigated by Shen et al. [26] using a turbocharged dual-fuel engine under high-load operation at 1500 rpm. An end-of-injection timing of 180^o^CA ATDC, combined with an energy replacement ratio between 4.6% and 8.9%, was identified as the optimal injection strategy. An assessment was conducted to determine how different methanol injection timings affect combustion characteristics and emission outcomes in marine diesel engine applications by Guo et al. [27]. Significant decreases in pollutant emissions, along with improved combustion efficiency, were achieved when methanol was injected at −30^o^CA, owing to better control of the in-cylinder combustion process.

Compared to simulations, experiments yield more precise and dependable results, but they also require a significant investment of time and cost. Along with this, simulations allow researchers to anticipate potential phenomena that may occur during experiments based on computational models and assumptions. This enables them to save time, cost, and resources by optimizing experimental design and reducing the need for extensive physical testing. Therefore, some researchers employ simulation tools like AVL Boost to forecast engine modifications before conducting actual engine testing. It is well known that AVL Boost is an effective program for modeling internal combustion engines. With the assistance of the AVL Boost software, the engine’s combustion model was developed, and it was able to predict the engine’s performance, emissions, and combustion characteristics. As a result, many researchers used the AVL Boost program to develop a virtual model of the diesel engine and investigated how different fuels affected the engine’s characteristics [28–37].

Hong et al. [28] used the AVL Boost software to investigate the characteristics of three diesel engines to propose a suitable diesel engine selection for firefighting in high-rise residential buildings, aiming to reduce costs and minimize environmental pollution. Li et al. [29] explored the influence of injection timing together with different diesel/ethanol/n-butanol blending proportions on diesel engine performance. Their results identified an optimal operating condition at a diesel fraction of 88.49% combined with an injection timing of 12.43^o^CA BTDC, which delivered the most favorable performance characteristics. Hong et al. [30,31] designed and optimized the drivetrain transmission ratio and operational load of a diesel firefighting engine. The results showed that a drivetrain transmission ratio of 1:1.2 at a 57% load could reduce fuel consumption by 8%. E et al. [32] and Nghia et al. [33] employed AVL Boost simulations to analyze how variations in biodiesel blending ratios (BR), injection timing, and injection pressure affect the performance and emission behavior of a diesel engine. Duong et al. [34] also employed AVL Boost software to compare the Vibe 2-Zone and Multiple Vibe 2-Zone combustion models, thereby proposing specific application scenarios for each model to optimize simulation efficiency and reduce time and resource consumption. Aldarwish et al. [35] used AVL FIRE software to determine the optimal injection timing and fuel injection quantity required to achieve the maximum brake mean effective pressure (BMEP). Nguyen et al. [36] also used AVL Boost to investigate the characteristics of a diesel engine operating on LPG–diesel dual fuel, and the results showed that a blend of 70% diesel and 30% LPG provided good performance while producing lower emissions. Ha et al. [37] numerically investigated the combustion, performance, and emission characteristics of a stationary diesel engine fueled with low ammonia–diesel blends (0–10% energy ratio) via AVL Boost software. The results indicated slight improvements in torque and power by 1%, significant reductions in NOx by 22% and CO emissions by 27%, and only minor variations in soot.

The aforementioned investigations primarily relied on experimental testing and numerical simulations to evaluate how variations in fuel blending proportions, injection pressure, and injection timing influence the performance, combustion behavior, and emission characteristics of diesel engines. From these findings, optimal combustion angles could be proposed to achieve benefits in terms of power output [21,22], fuel efficiency [18–20,22], or emission reduction [18–21]. Furthermore, prior research by the authors employed both the two-zone Vibe and multi–two-zone Vibe combustion models to identify the crank angles that optimize diesel engine performance, specifically targeting peak power output while reducing torque demand under various premixed ratios, combustion durations (CD), and loads [38,39]. Together with this, in studies on methanol–diesel fuel blends in internal combustion engines, there have been numerous investigations on optimizing injection timing with the aim of improving engine efficiency [24–27] and exhaust emissions [23–27] under different engine loads or operating speeds. While previous studies have investigated ignition or injection timing optimization, they have generally focused on individual parameters such as injection timing, injection pressure, pilot injection, or fuel properties in conventional diesel or methanol–diesel engines. In contrast, the present study addresses the combined effects of blending ratio, CD, and engine load on ignition timing, which has not been systematically explored. Furthermore, this study determines the optimal ignition timings (IT) for achieving maximum power output and minimum BSFC across a range of methanol–diesel blends under varying load and CD conditions. Beyond identifying optimal ITs, this study provides comprehensive predictive models for optimal IT across a wide range of methanol–diesel blends, combustion durations, and load conditions. To achieve greater predictive accuracy, an enhanced modeling strategy was implemented in which critical combustion variables – such as CD and m – were systematically tuned in accordance with the BR. The parameter adjustments were guided by earlier experimental observations and subsequently verified against calibration datasets to maintain strong model reliability. Unlike previous investigations, this methodology provides a more realistic and precise characterization of dual-fuel engine performance. In the current study, the term ignition timing (IT) is defined as the start of combustion of diesel fuel in the combustion chamber.

To address this objective, a simulation model of the Vikyno RV125 engine was developed using AVL Boost to analyze the impacts of varying methanol–diesel fuel proportions and IT angles on engine performance, including power, torque, and BSFC. The findings are then used to determine the ideal ITs for achieving the highest power and lowest BSFC at different BRs and CD under engine loads of 85, 70, and 50%. The study also developed predictive models for the ideal ITs based on BRs and CD at these load conditions. This study focused on this topic because BRs and CDs are interdependent parameters that can be determined experimentally. In practice, identifying the optimal IT requires conducting numerous test cases to evaluate their combined effects. However, by utilizing this predictive model, experiments are carried out to determine the CDs [40] and BRs, and then using the predictive model to forecast the ideal IT after substituting these parameters.

The objectives of this investigation include: (1) examine the impact of various ITs, CDs and BRs on the performance characteristics of the diesel engine at 50, 70, and 85% loads, (2) establish the most favorable IT conditions for maximizing engine power and reducing BSFC, (3) develop a predictive model for the ideal ITs based on the relationship between mixture ratios, CDs, and loads. This study provides results on the effects of different BRs, ITs, CDs, and loads on the engine, allowing identification of the factors that most significantly influence the engine’s performance characteristics. In addition, manufacturers can calibrate the engine using the established predictive model to determine the ideal ITs based on adjustable parameters such as engine type, CDs, BRs, and load conditions, depending on the usage scenario, instead of conducting multiple physical experiments. From the optimal ITs, manufacturers can predict the appropriate injection timing angles based on the ignition delay. This enables the engine to attain the lowest BSFC and the greatest power, while improving operational efficiency, saving fuel, and providing economic benefits.

### Simulation setup

**Fig 1:**
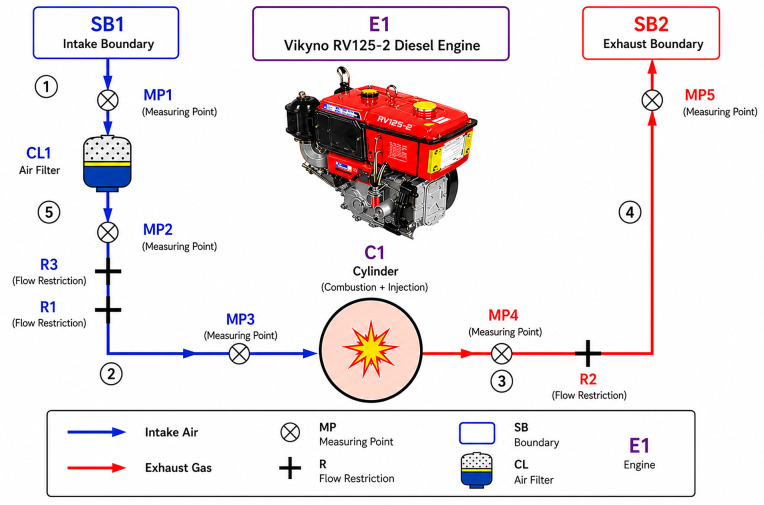
The AVL Boost engine block schematic‌‌.

The Vikyno RV-125 diesel direct injection engine was used in this investigation. This study utilizes an engine configured with one cylinder operating on a four-stroke cycle. Generating 12.5 HP at 2400 rpm and attaining its highest torque of 4.04 kgf·m at 1800 rpm, the engine demonstrates strong operational capability in the low-to-mid speed region. Its geometric specifications comprise a 94 mm bore and a 90 mm stroke, and it operates under a relatively elevated compression ratio of 18:1, a common feature of compression-ignition systems that facilitates effective fuel combustion. Additionally, the engine displacement is 624 cm³, representing a small-displacement configuration suitable for experimental investigation and controlled performance evaluation. To forecast the ideal ignition timing (IT), the research engine model was developed in the AVL Boost environment, as seen in Fig 1.

The portions of the engine employed in the experiment are described by the components of the simulation model, which served as a means of examining the properties of the engine components. The engine’s intake and exhaust systems, or SB1 and SB2, are the system borders that symbolize the system’s limitations. One cylinder (C1) is also the most important component of the model. The air purifier in the system was element CL1. Element E1 is used to specify the overall simulation parameters, including ignition timing (IT), the number of crank rotations, and other global conditions. Meanwhile, elements R1, R2, and R3 are assigned to represent flow restrictions, accounting for pressure losses occurring along the intake and exhaust ducts (pipes 1–5). The flow characteristics were calculated using the measurement elements (MP1–MP5) in the intake and exhaust pipes, such as the air mass flow rate, the flow velocity, and the temperature. The parameters of the schematic diagram are summarized in Table 1.

**Table 1:** Summary of schematic diagram elements.

Element	Component Name	Function / Description
SB1	Intake system boundary	Defines the inlet boundary of the system and represents the intake domain limit
SB2	Exhaust system boundary	Defines the outlet boundary of the system and represents the exhaust domain limit
C1	Cylinder	Main component where combustion and energy conversion occur
CL1	Air filter	Represents the air purification process before air enters the intake system
E1	Global parameter element	Specifies overall simulation settings such as ignition timing (IT), crank rotations, etc.
R1, R2, R3	Flow restrictions	Model pressure losses along intake and exhaust ducts (pipes 1–5)
MP1–MP5	Measurement points	Used to evaluate flow characteristics (mass flow rate, velocity, temperature)

As illustrated in Fig 1, the simulation model represents a single-cylinder diesel engine system. The intake and exhaust boundaries are defined by SB1 and SB2, respectively. Air enters the system through the intake boundary (SB1), passes through the air filter (CL1), and flows along the intake pipes (pipes 1–2). The intake air is provided as an experimentally measured input and is implemented in the simulation model at the air filter element (CL1). The flow then reaches the cylinder (C1), which is the core component where the thermodynamic processes, including intake, compression, fuel injection, combustion, expansion, and exhaust, occur based on the crank angle. Here, the injected fuel quantity is controlled at the cylinder element (C1), and this fuel injection amount is also determined experimentally. The applied combustion model enables the prediction of key engine performance parameters, including power, torque, brake specific fuel consumption (BSFC), as well as detailed combustion characteristics of the tested fuels. After combustion, the exhaust gases are discharged through the exhaust pipe (pipes 3–4) and exit the system via the exhaust boundary (SB2). Flow restrictions (R1, R2, and R3) are incorporated into the intake and exhaust ducts to account for pressure losses along the flow path. The global simulation parameters, such as ignition timing (IT), engine speed, and number of crank rotations, are defined by element E1. To evaluate the flow behavior within the system, measurement points (MP1–MP5) are positioned along the intake and exhaust pipes. These points are used to determine key flow characteristics, including air mass flow rate, flow velocity, and temperature distribution. Furthermore, the AVL BOOST model operates based on one-dimensional gas dynamics, solving the conservation equations of mass and energy along the intake and exhaust systems as a function of crank angle. This approach enables accurate prediction of engine performance and combustion characteristics.

The simulation model was designed to replicate the general properties of the original diesel engine. Simulations were conducted at load conditions of 50, 70, and 85% across speeds of the engine from 900 to 2400 rpm in 300 rpm increments, while evaluating six fuel blends: D100, D90M10, D80M20, D70M30, D60M40, and D50M50. The combustion duration (CD) was changed according to the change of the blending ratio (BR) [41,42]. To assess the impact of various ITs and BRs on the power, the IT values are varied at 2400 rpm with 85, 70, and 50% load for each case of the BR, load, and CD. To evaluate brake specific fuel consumption (BSFC), a range of IT settings was examined at five separate engine speeds, spanning 900–2400 rpm, under 85, 70, and 50% load conditions. These variations were applied for all combinations of BR, engine load, and CD. Ultimately, the predictive models were developed based on the peak power obtained at 2400 rpm and the minimum BSFC area, which was calculated by integrating BSFC values over the engine speed range of 1500–2400 rpm. In this study, the optimal IT of the Vikyno RV-125 diesel engine is investigated under two operating conditions: maximum power and fuel economy. Typically, achieving maximum power requires the engine to operate at its highest speed. Therefore, a speed of 2400 rpm was selected to determine the optimal IT under high-load conditions. For the minimum BSFC condition, the analysis was conducted over a speed range of 900–2400 rpm. This range was chosen because stationary engines are commonly operated between the speed corresponding to maximum torque and that of maximum power. The term ignition timing (IT) in this work denotes the start of combustion of diesel fuel in the combustion chamber.

This study examined six different fuel types, including pure diesel which was designated as D100; and methanol blended with diesel at five different volume ratios of 10, 20, 30, 40, and 50%, were labeled as D90M10, D80M20, D70M30, D60M40, and D50M50, respectively. The physical characteristics of the fuel are displayed in Table 2. Finally, the quantity of fuel that is delivered into the cylinder, as simulated in the software, at different BRs and loads, is presented in Table 3. Additionally, the properties of methanol–diesel fuel blends are presented in Table 4.

**Table 2:** The properties of methanol and diesel fuels [43].

Specification	Diesel	Methanol
Liquid density (kg/m^3^)	850	792
Low heating value (kJ/kg)	42800	21118
Stoichiometric A/F ratio	14.7	6.47
Molar mass (kg/kmol)	100.21	32.04
Carbon/total mass ratio	0.84	0.37
Oxygen/total mass ratio	0	0.50
Heat of evaporation (kJ/kg)	275	1101

**Table 3:** Quantity of fuel delivered into the cylinder per cycle at different loads and methanol–diesel blending ratios.

Blending ratios	Fuel per cycle (g/cycle)		
	85% engine load	70% engine load	50% engine load
D100	0.028	0.024	0.019
D90M10	0.029	0.026	0.020
D80M20	0.030	0.027	0.021
D70M30	0.031	0.028	0.022
D60M40	0.033	0.030	0.023
D50M50	0.037	0.032	0.026

**Table 4:** Properties of methanol–diesel blends [43].

Specification	D100	D90M10	D80M20	D70M30	D60M40	D50M50
Liquid density (kg/m^3^)	850	844.2	838.4	832.6	826.8	821
Low heating value (kJ/kg)	42800	40793.4	38728.2	36634.2	34510.9	32357.5
Stoichiometric A/F ratio	14.7	14.35	13.53	12.69	11.84	10.97
Molar mass (kg/kmol)	100.21	83.53	71.48	62.35	55.21	49.46
Carbon/total mass ratio	0.84	0.796	0.751	0.707	0.661	0.615
Oxygen/total mass ratio	0	0.047	0.0943	0.142	0.191	0.241
Heat of evaporation (kJ/kg)	275	352.49	431.06	510.72	591.49	673.41

### Thermodynamic model

The thermodynamic condition of the in-cylinder working medium is determined from the energy conservation applied to the engine cylinder. The first thermodynamic equation [43–45] states the following:


$$\begin{array}{c} \frac{d\left(m_{c}\cdot u\right)}{d\alpha }=-p_{c}\cdot \frac{dV}{d\alpha }+\frac{dQ_{F}}{d\alpha }-\sum \frac{dQ_{w}}{d\alpha }-h_{BB}\cdot \frac{dm_{BB}}{d\alpha }+\sum \frac{dm_{i}}{d\alpha }\cdot h_{i}-\sum \frac{dm_{e}}{d\alpha }\cdot h_{e}-q_{ev}\cdot f\cdot \frac{dm_{ev}}{dt} \end{array}$$ (1)

The variations in the cylinder mass are evaluated by accounting for the net balance between the incoming and outgoing mass flows:


$$\begin{array}{c} \frac{dm_{c}}{d\alpha }=\sum \frac{dm_{i}}{d\alpha }-\sum \frac{dm_{e}}{d\alpha }-\frac{dm_{BB}}{d\alpha }+\frac{dm_{ev}}{dt} \end{array}$$ (2)

In accordance with the AVL BOOST theoretical framework [43], Eq (1) has been reformulated to clearly separate boundary work, heat transfer, and mass-related energy exchange, including the effect of fuel evaporation:

- The rate of change of energy within the cylinder

$$\begin{array}{c} \frac{d\left(m_{c}\cdot u\right)}{d\alpha } \end{array}$$ (3)

- Work term represents the mechanical work exchanged between the in-cylinder gas and the piston

$$\begin{array}{c} \dot{W}=-p_{c}\frac{dV}{d\alpha } \end{array}$$ (4)

- Heat transfer terms

$$\begin{array}{c} \dot{Q}=\frac{dQ_{F}}{d\alpha }-\sum \frac{dQ_{w}}{d\alpha } \end{array}$$ (5)

- Mass transfer and enthalpy terms

$$\begin{array}{c} {\dot{m}}_{energy}=-h_{BB}\frac{dm_{BB}}{d\alpha }+\sum \frac{dm_{i}}{d\alpha }h_{i}-\sum \frac{dm_{e}}{d\alpha }h_{e} \end{array}$$ (6)

- Fuel evaporation

$$\begin{array}{c} {\dot{Q}}_{evap}=-q_{ev}\cdot f\cdot \frac{dm_{ev}}{dt} \end{array}$$ (7)

where *h*_*BB*_ refers to the specific enthalpy of blow-by (J/kg). *f* characterizes the fraction of evaporation heat associated with the cylinder charge. *q*_*ev*_ corresponds to the latent heat of fuel evaporation (J). *m*_*ev*_ denotes the mass of fuel that undergoes evaporation (kg). *m*_*i*_ represents the mass component entering the cylinder (kg). *h*_*i*_ is the specific enthalpy of the incoming mass (J/kg). *h*_*e*_ refers to the specific enthalpy of the mass leaving the cylinder (J/kg). *m*_*c*_ denotes the total mass contained within the cylinder (kg). *m*_*e*_ indicates the mass component ejected from the cylinder (kg). *m*_*BB*_ represents the blow-by mass (kg). *u* is the specific internal energy (J/kg). *p*_*c*_ cylinder pressure (bar). V corresponds to the volume in the cylinder (m^3^). *Q*_*F*_ refers to the energy of fuel (J). *Q*_*w*_ is the heat loss through the wall (J). *α* is the angle of crankshaft (deg).

### Combustion model

In this work, the combustion model was based on the Vibe 2-zone. The heat release properties displayed in calculations 3, 4, and 5 may be easily described by the Vibe function [43]:


$$\begin{array}{c} \frac{dx}{d\alpha }=\frac{a}{\Delta \alpha_{c}}\cdot \left(m+1\right)\cdot y^{m}\cdot exp\left[-a\cdot y^{\left(m+1\right)}\right] \end{array}$$ (8)

$$\begin{array}{c} dx=\frac{dQ}{Q} \end{array}$$ (9)

$$\begin{array}{c} y=\frac{\alpha -\alpha_{0}}{\Delta \alpha_{c}} \end{array}$$ (10)

where *Q*, *α*, *α*_*0*_, Δ*α*_*c*_, *m*, and *a* are the total thermal input from fuel, crank angle, start of combustion, combustion duration, shape parameter, and Vibe parameter, respectively.

**Fig 2:**
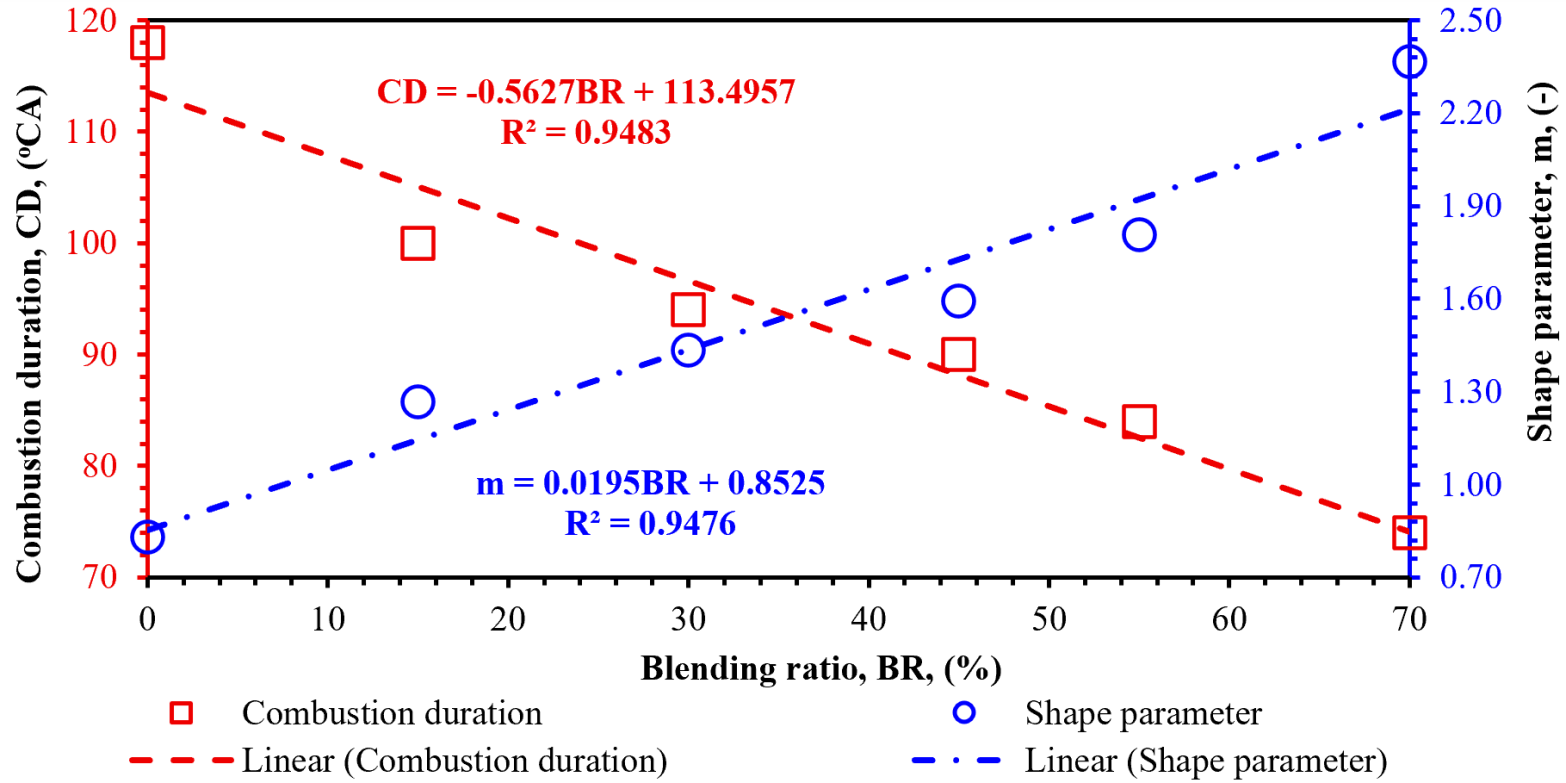
The predictive model was developed to predict the shape parameter, m, and combustion duration, CD, from experimental results with respect to the blending ratio, BR [41].

Vibe 2-Zone is defined by several key parameters, including CD, the shape parameter ‘m,’ the start of combustion, and the vibe parameter ‘a.’ For the diesel engine examined in this study, a value of 6.9 was assigned to the Vibe coefficient to ensure complete combustion, while the shape parameter was adjusted in response to changes in the diesel–methanol mixture proportion. The study applied the reference [41] to determine the shape parameter m and CD of the diesel and other BRs. Fig 2 illustrates the variation of the shape parameter m and CD with different methanol BRs in diesel, based on the experimental study [41]. Using these values, predictive formulas for the shape parameter and CD as functions of methanol percentage were established, allowing the calculation of shape parameter and CD values for methanol BRs ranging from 0 to 50%.

Based on Fig 2, every 10% increase in the methanol blending ratio results in a 16.51% increase in the shape parameter. Therefore, the shape parameter m for each BR is determined, and this value is kept constant at 50, 70, and 85 loads. In the case of 100% diesel fuel, the shape parameter was selected as m = 0.6 according to the recommendation of AVL Boost for diesel engines [43]. Subsequently, the shape parameter m values for the other BRs were determined based on the m value of D100. The shape parameter m takes values of 0.60, 0.70, 0.81, 0.95, 1.11, and 1.29 for D100, D90M10, D80M20, D70M30, D60M40, and D50M50, respectively.

**Fig 3:**
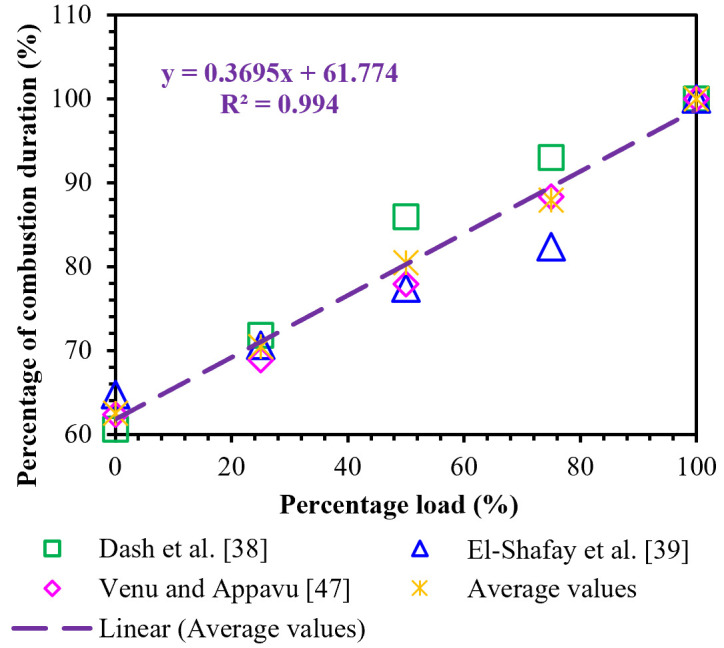
Predictive equation for the percentage of combustion durations as a function of engine load for D100.

From the literature [41,46], the higher the mass fraction of the methanol in the methanol/diesel blend, the shorter the CD. Based on Fig 2, for every 10% increase in the methanol–diesel BR, the CD decreases by 5.54%. From the CD of the D100 at 85% load (60^o^CA, 80^o^CA, 100^o^CA and 120^o^CA), the CD of other fuel mixtures is estimated. From the literature [38,39,47], the CD of the pure diesel at five loads (0, 25, 50, 75 and 100%) was obtained from experiments. From these CD values, the average CDs at different loads were calculated, and a predictive model between CDs and loads was established. Fig 3 shows the experimental CDs with average values from the reference [38,39,47] and the predictive model to predict CDs at different loads for simulation, respectively. Using the experimental data reported by Dash et al. [38], El-Shafay et al. [39], and Venu and Appavu [47], the CD decreases as the engine load is reduced from 100 to 0%. As engine load increases, combustion duration becomes longer due to higher fuel consumption, a richer mixture, reduced oxygen concentration, and deteriorated air–fuel mixing, all of which slow down the combustion process. Similar results have also been reported in other studies [48–51]. Accordingly, the average CD values (average values) derived from these experimental datasets at different load conditions were calculated and used to develop a predictive model of CD as a function of the load percentage, as shown in Fig 3. From Fig 3, when the load decreases, the CD also decreases, and based on the established predictive model, a 10% reduction in load results in an approximate 5% decrease in CD. Therefore, the CD values for BRs from 0 to 50% at loads of 50, 70, and 85% are presented in Table 5.

**Table 5:** The combustion duration at different diesel/methanol blending ratios at 85, 70, and 50% engine loads.

Fuel blending ratio	Combustion duration (^o^CA)											
	85% engine load				70% engine load				50% engine load			
D100	60	80	100	120	56	75	94	113	52	69	86	104
D90M10	57	76	94	113	53	71	89	107	49	65	82	98
D80M20	54	71	89	107	50	67	84	101	46	62	77	92
D70M30	51	67	84	101	48	63	79	95	44	58	73	87
D60M40	48	64	80	96	45	60	75	90	41	55	69	82

Two zones, each of which is spatially homogenous, are employed for the combustion process. The burnt and unburned zones are the two areas. The following equation presents the burned charge and unburned charge, respectively [43]:


$$\begin{array}{c} \frac{d\left(m_{b}u_{b}\right)}{d\alpha }=-p_{c}\frac{dV_{b}}{d\alpha }+\frac{dQ_{F}}{d\alpha }-\sum \frac{dQ_{Wb}}{d\alpha }+h_{u}\frac{dm_{b}}{d\alpha }-h_{BB,b}\frac{dm_{BB,u}}{d\alpha }\; \end{array}$$ (11)

$$\begin{array}{c} \frac{d\left(m_{u}u_{u}\right)}{d\alpha }=-p_{c}\frac{dV_{u}}{d\alpha }-\sum \frac{dQ_{Wu}}{d\alpha }-h_{u}\frac{dm_{b}}{d\alpha }-h_{BB,u}\frac{dm_{BB,u}}{d\alpha } \end{array}$$ (12)

$$\begin{array}{c} \frac{dV_{b}}{d\alpha }+\frac{dV_{u}}{d\alpha }=\;\frac{dV}{d\alpha } \end{array}$$ (13)

$$\begin{array}{c} V_{b}+V_{u}=V \end{array}$$ (14)

where the variables *m*_*b*_ and *m*_*u*_ refer to the masses of the burned and unburned mixtures (kg), respectively. *α* corresponds to the crank angle. *h*_*BB,u*_ represents the specific enthalpy carried away by blow-by gases (unburned zone) (J/kg). *h*_*BB,b*_ represents the specific enthalpy carried away by blow-by gases (burned zone) (J/kg). *Q*_*F*_ denotes the total heat released by fuel combustion (J). *V*_*b*_ and *V*_*u*_ are the volumes of the burned and unburned zone (m^3^), respectively. *V* is the total cylinder volume (m^3^). *u*_*b*_ and *u*_*u*_ are the specific internal energy of burned and unburned gases (J/kg), respectively. *Q*_*Wu*_ represents the heat loss from the unburned zone to the wall (J). *Q*_*Wb*_ represents the heat loss from the burned zone to the wall (J). *p*_c_ denotes the cylinder pressure (bar). *h*_u_ is the specific enthalpy of the unburned mixture (J/kg). *m*_*BB,u*_ is the blow-by mass from the unburned zone (kg).

### Model of heat transfer

The heat flux transferred to the combustion chamber boundaries, namely the cylinder head, piston crown, and cylinder liner, is evaluated using the equation given below:


$$\begin{array}{c} Q_{W}=A_{i}\alpha_{w}(T_{c}-T_{wi}) \end{array}$$ (15)

where *Q*_*W*_*, T*_*c*_*, T*_*wi*_*, A*_*i*_*,* and *α*_*w*_, are heat flow loss through walls, gas temperature within the cylinder, wall temperature, surface area, and convective heat transfer coefficient, respectively

The method of heat transmission within the diesel engine’s cylinder is quite intricate. The Woschni correlation, developed in 1978, is widely applied for estimating the heat transfer coefficient in diesel engines. Due to its ease of use and sufficient accuracy, this formula is employed in this investigation. For the 1978 Woschni model, the coefficient of heat transmission is calculated using the following equation:


$$\begin{array}{c} \alpha_{w}=130\cdot D^{-0.2}\cdot \overset{0.8}{\underset{c}{p}}\cdot \overset{-0.53}{\underset{c}{T}}\cdot {\left[C_{\text{1}}\cdot C_{m}+C_{\text{2}}\cdot \frac{V_{D}\cdot T_{c,1}}{p_{c,1}\cdot V_{c,1}}\cdot \left(p_{c}-p_{c,0}\right)\right]}^{0.8} \end{array}$$ (16)

where the symbols *V*_*D*_ and *D* indicate the engine displacement volume (m^3^) and cylinder bore diameter (m), respectively. In this formulation, *C*_*m*_ and *C*_*u*_ refer to the mean and circumferential piston speeds (m.s^-1^). The parameters *C*_*1*_ and *C*_*2*_ correspond to the gas velocity coefficient and the model constant, respectively, with *C*_*1*_ *= 2.28 + 0.308C*_*u*_*/C*_*m*_, and *C*_*2*_ *= 0.00342* for direct injection engine. Furthermore, V_c*,1*_ and *p*_*c,1*_ describe the cylinder volume (m^3^) and pressure (bar) at the moment of inlet valve closure, while *V* and *p*_*c,0*_ represent the actual instantaneous cylinder volume (m^3^) and the reconstructed (reversed) cylinder pressure (bar). *p*_*c*_ denotes the in-cylinder pressure (bar). *α*_*w*_ is the temperature-transfer efficiency. *p*_*c,0*_ denotes the cylinder pressure of the engine (bar). *T*_*c,1*_ refers to the temperature of the cylinder at the intake valve closes (K). *T*_*c*_ is the cylinder temperature (K).

### Model validation

In the authors’ previous study [52], experiments were conducted on a Vikyno RV-125 engine operated under three load conditions of 85, 70, and 50% to measure key performance parameters, including actual output power, brake torque, fuel mass flow rate, and intake air flow rate. Detailed information regarding the experimental apparatus can be found in reference [52]. In addition, to ensure experimental reproducibility, information on experimental equipment, uncertainty, and margin of error is provided in the S1 File. Model validation and result verification were performed using the data obtained at the 50% and 85% load conditions. Using the experimentally measured fuel consumption and intake air flow rates as inputs, AVL Boost simulations were performed to estimate the indicated mean effective pressure (*IMEP*_*simul*_), friction mean effective pressure (*FMEP*_*simul*_), and brake mean effective pressure (*BMEP*_*simul*_). Eq (17) can be used to determine the engine’s simulated torque based on the BMEP [53]:


$$\begin{array}{c} Tor_{simul}=\frac{BMEP_{simul}.V_{D}}{4\pi } \end{array}$$ (17)

Simulation power is obtained using the following formula [53]:


$$\begin{array}{c} P_{simul}=2\pi Tor_{simul}N \end{array}$$ (18)

where N is engine speed (rpm)

**Fig 4:**
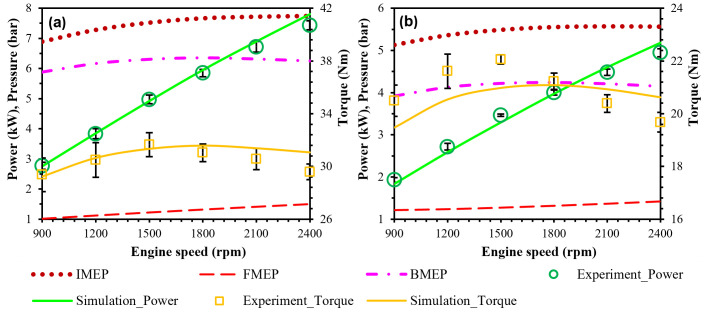
Comparison of IMEP, FMEP, BMEP, and simulated versus experimental torque and power at the different engine speeds under (a) 85% load and (b) 50% load.

The validity of the AVL Boost model was assessed by comparing the simulated torque and power with corresponding experimental results from Ref. [52]. Fig 4 presents the variations of IMEP, FMEP, BMEP, as well as simulated and measured torque and power, as functions of engine speed under a 50% load condition. Within the scope of this study, the results indicate a strong correlation between the simulation predictions and the experimental data. The numerical model exhibits good accuracy and reliability when compared to actual engine performance, as evidenced by the simulation AVL model’s maximum error of less than 5%. At 85% load, the mean absolute percentage errors in power and torque between the simulation and experiment are 1.84 and 1.90, while the maximum errors are 4.97% and 4.73%, respectively. At 50% load, the corresponding mean absolute percentage errors are 3.77% and 3.81%, while the maximum errors are 4.93% and 4.96%.

## Results and discussions

### Influence of ignition timing and fuel blending ratio on power at different engine loads

**Fig 5:**
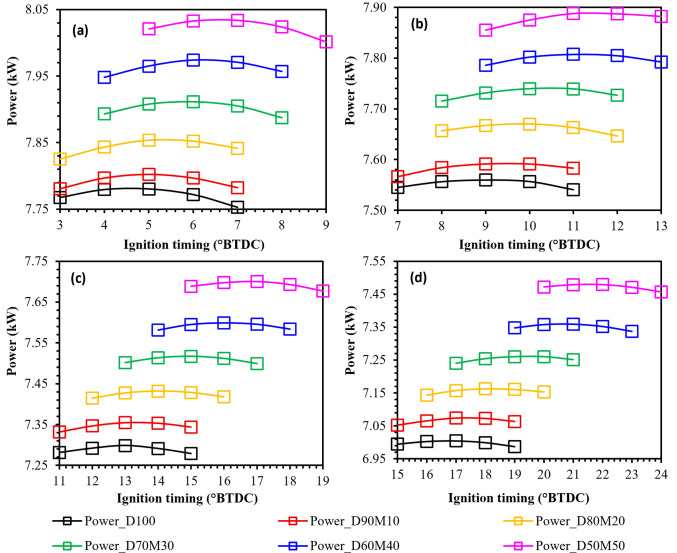
Engine power variation with ignition timing at 2400 rpm and 85% load with different blending ratios at (a) CD60, (b) CD80, (c) CD100, (d) CD120.

**Fig 6:**
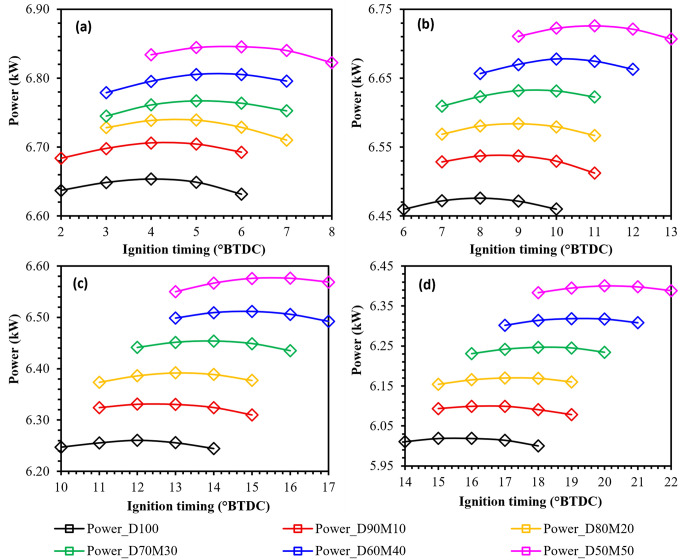
Engine power variation with ignition timing at 2400 rpm and 70% load with different blending ratios at (a) CD60, (b) CD80, (c) CD100, (d) CD120.

**Fig 7:**
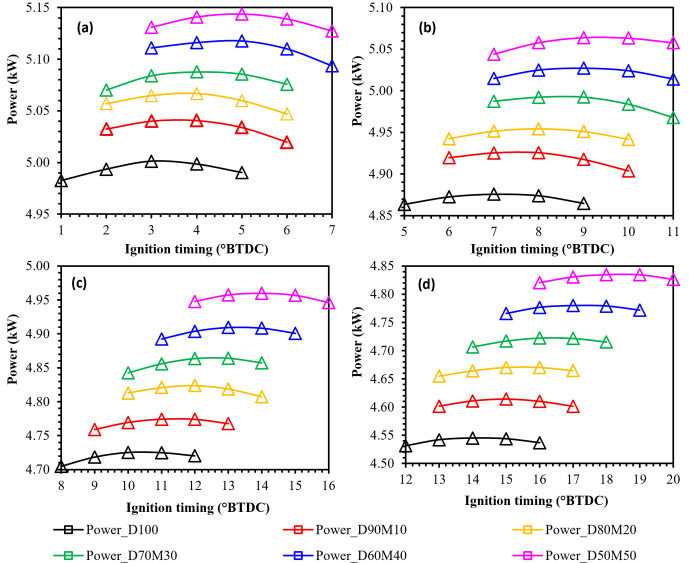
Engine power variation with ignition timing at 2400 rpm and 50% load with different blending ratios at (a) CD60, (b) CD80, (c) CD100, (d) CD120.

Figs 5–7 depict the effect of varying ignition timings (IT) on engine power across six fuel blends—D100, D90M10, D80M20, D70M30, D60M40, and D50M50—considering four combustion durations of 60^o^CA (CD60), 80^o^CA (CD80), 100^o^CA (CD100), and 120^o^CA (CD120) at engine loads of 85, 70, and 50%, respectively. When IT is advanced, power first reaches its peak, then drops. Power increases first because the peak pressure occurs closer to the piston’s top dead center. However, delaying IT results in a corresponding delay in combustion, leading to a rise in pressure primarily during the rapid expansion phase of the cylinder. As a result, the engine power value decreases along with the mean effective pressure [54]. Increasing the blending ratio (BR) increases power. Methanol burns faster than diesel, leading to higher pressure near the end of combustion. Additionally, methanol contains OH groups that promote better combustion due to its higher oxygen content. Methanol also has a latent heat of vaporization four times greater than that of diesel, allowing it to absorb more heat from the engine during evaporation. This phenomenon lowers the in-cylinder temperature and improves volumetric efficiency. Although methanol has a lower energy content than diesel, its simple chemical structure—with only one carbon atom compared with diesel’s long carbon chains (C₁₂–C₂₂)—allows for a more complete combustion process as the methanol ratio increases. These findings are consistent with previous studies [55–59]. At 85% load and CD 60^o^CA, the difference in maximum power between D100 and D50M50 is 0.253 kW, corresponding to an increase of 3.254%. In addition, increasing the methanol blending ratio in the fuel mixture leads to an increase in the peak heat release rate (HRR). An increase in HRR at elevated methanol blending ratios is attributed to the oxygenated characteristics of methanol, which enhance the kinetically controlled combustion stage in the diffusion phase, leading to improved heat release and overall combustion efficiency [60,61]. A longer ignition delay is induced by the low cetane number of methanol, leading to greater fuel accumulation in the premixed phase, which subsequently elevates combustion temperature and improves thermal efficiency [42]. As CD increases from 60^o^CA to 120^o^CA, the maximum power decreases. Longer combustion duration delays combustion into the expansion stroke, increasing heat losses and lowering combustion efficiency. An increase in CD also reduces both peak temperature and peak pressure, which contributes to the decrease in power [62]. At 85% load, the maximum power of D50M50 decreases from 8.034 kW on CD 60°CA to 7.479 kW at CD 120^o^CA. In addition, engine power increases with increasing load. An increase in engine load leads to a higher fuel injection quantity, resulting in enhanced heat release and elevated in-cylinder pressure. The higher in-cylinder temperature at elevated load conditions facilitates improved fuel evaporation [63] Poorer combustion is observed at low loads due to the cooling effect and leaner air–fuel mixture, which consequently results in reduced combustion efficiency [55]. At CD 60^o^CA, the maximum power of D50M50 decreases from 8.034 kW at 85% load to 6.846 kW at 70% load and further drops to 5.144 kW at 50% load.

### The predictive model for maximum power at 50, 70, and 85% engine loads

**Fig 8:**
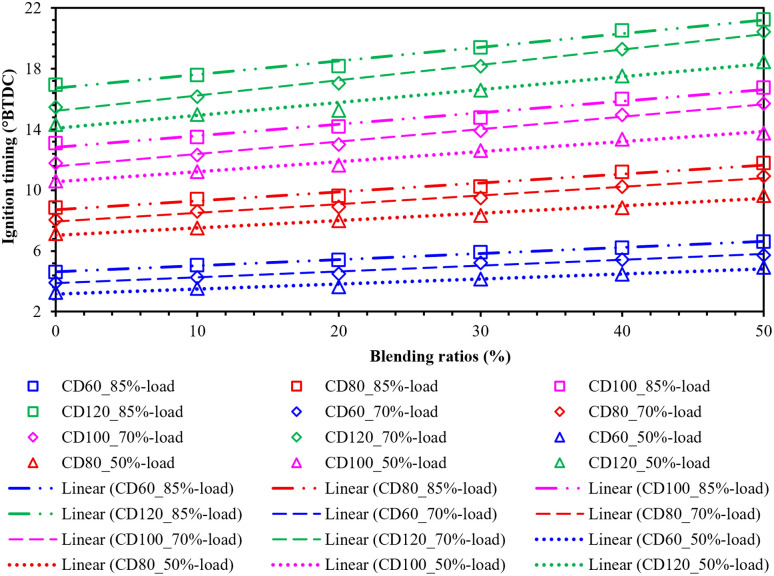
The developed model predicts the ignition timing that yields peak power with different operating conditions, specifically at engine loads of (a) 85%, (b) 70%, and (c) 50%.

Fig 8 shows the optimal ITs for maximum power at engine loads of 85, 70, and 50%, and 2400 rpm under four CDs. The higher the methanol percentage in the blend, the greater the delay in achieving the optimal IT. Compared with conventional diesel, methanol exhibits a higher ignition temperature, an increased latent heat of vaporization, and a lower cetane number [58]. During vaporization, methanol draws considerable heat from the combustion chamber, thereby lowering the temperature increase inside the cylinder. This reduction in temperature slows down the ignition process and leads to a longer ignition delay. Therefore, increasing the methanol fraction in the fuel mixture leads to a longer ignition delay, requiring the IT to be advanced to achieve optimal power output. The difference in the optimal IT for maximum power at 2400 rpm across the three load conditions (50, 70, and 85%) between the two BRs, D100 and D50M50, ranges from a minimum of 2^o^CA to a maximum of 5^o^CA. As the CD increases, the optimal IT also becomes earlier. A longer CD reduces both the in-cylinder temperature and pressure while increasing the time required to complete combustion. Conversely, a shorter CD shifts the combustion process closer to the ideal Otto cycle by improving combustion efficiency and HRR [64]. The optimal IT required to achieve maximum power at CD120^o^CA is earlier than at CD 60^o^CA, with the difference ranging from 11^o^CA to 15^o^CA.

Finally, optimal IT also advances as load increases. With increasing engine load, more fuel is supplied to the cylinder, which increases pressure and temperature inside the combustion chamber and consequently shortens the ignition delay [65]. During combustion, the radicals OH and HO₂ are formed, and methanol influences the balance of their concentrations in the reaction regions. Because methanol enhances the formation of H₂O₂—a relatively stable radical at lower in-cylinder temperatures and pressures—its presence leads to an increase in ignition delay at low engine loads. Moreover, variations in methanol content significantly influence combustion behavior under these conditions [66]. Because higher engine loads produce elevated in-cylinder temperature and pressure, the influence of fuel properties on combustion diminishes. These conditions promote H₂O₂ formation and accelerate the fuel–air reaction process [67]. The ideal IT corresponding to peak power differs by approximately 1- 4^o^CA when comparing 50 and 85% engine loads. The results indicate that CD exerts the dominant effect on the IT required to maximize power output, with the methanol BR exerting a secondary influence, and engine load contributing the least.

Furthermore, this work proposes a predictive model to estimate the ideal IT for achieving maximum power. The graph demonstrates a linear relationship among the three parameters: load, CD, and BR. Furthermore, a predictive model for estimating the optimal IT corresponding to maximum power as a function of BR at different CDs and loads was developed, and the coefficient of determination (R²) was presented in Table 6.

**Table 6:** Prediction model for optimal IT achieving maximum power as a function of blending ratio.

CD (^o^CA)	Predictive optimal ignition timing model		
	85% engine load	70% engine load	50% engine load
60	IT = 0.0397BR + 4.6381R² = 0.996	IT = 0.0383BR + 3.8795R² = 0.9743	IT = 0.0331BR + 3.1719R² = 0.9686
80	IT = 0.0588BR + 8.7167R² = 0.9694	IT = 0.0572BR + 7.939R² = 0.983	IT = 0.0485BR + 7.0362R² = 0.9785
100	IT = 0.0759BR + 12.815R² = 0.9755	IT = 0.0815BR + 11.575R² = 0.9906	IT = 0.0663BR + 10.545R² = 0.9882
120	IT = 0.09BR + 16.717R² = 0.9856	IT = 0.1009BR + 15.236R² = 0.9924	IT = 0.0847BR + 14.075R² = 0.9718

### Influence of ignition timing and fuel blending ratio on engine brake-specific fuel consumption at different engine loads

**Fig 9:**
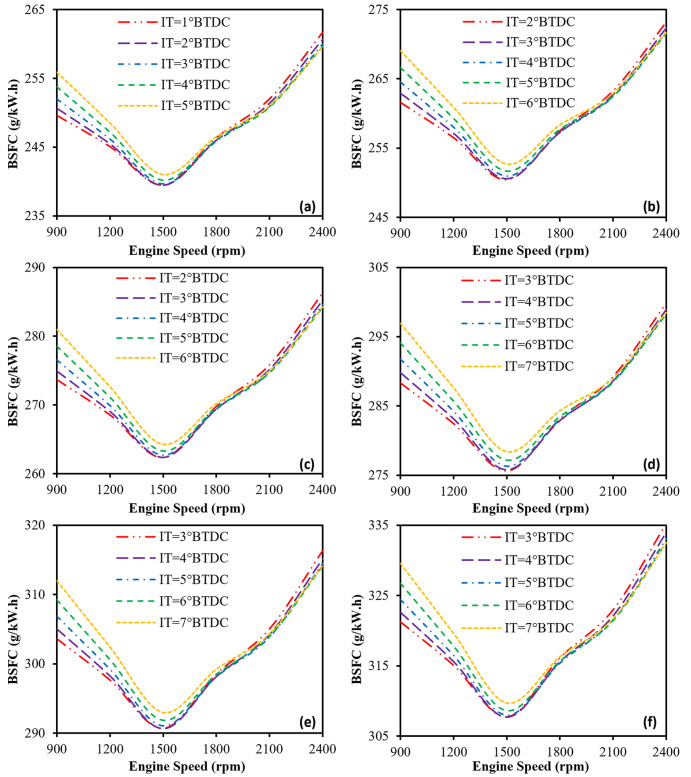
Effect of ignition timing on BSFC at 85% engine load for various blending ratios at CD60 of (a) D100, (b) D90M10, (c) D80M20, (d) D70M30, (e) D60M40, and (f) D50M50.

**Fig 10:**
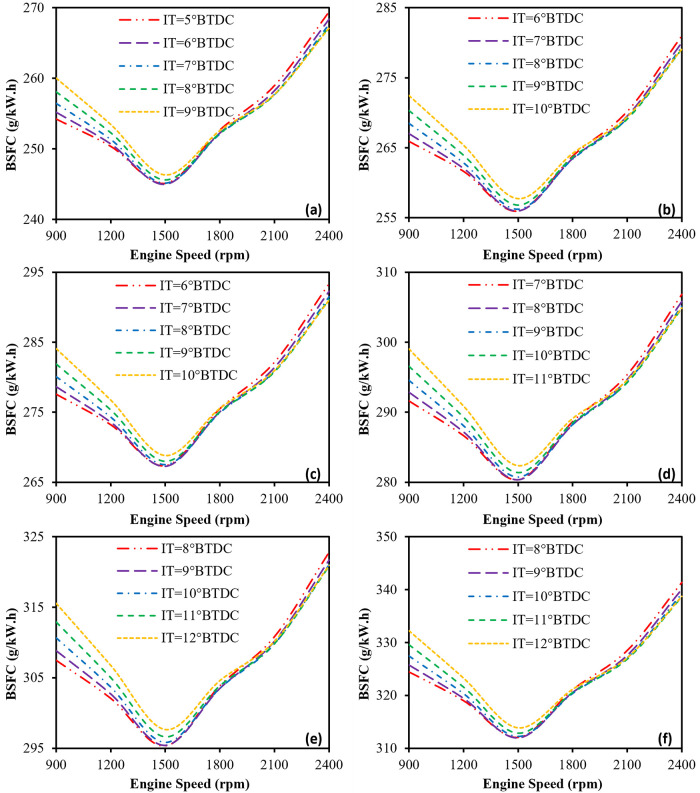
Effect of ignition timing on BSFC at 85% engine load for various blending ratios at CD80 of (a) D100, (b) D90M10, (c) D80M20, (d) D70M30, (e) D60M40, and (f) D50M50.

**Fig 11:**
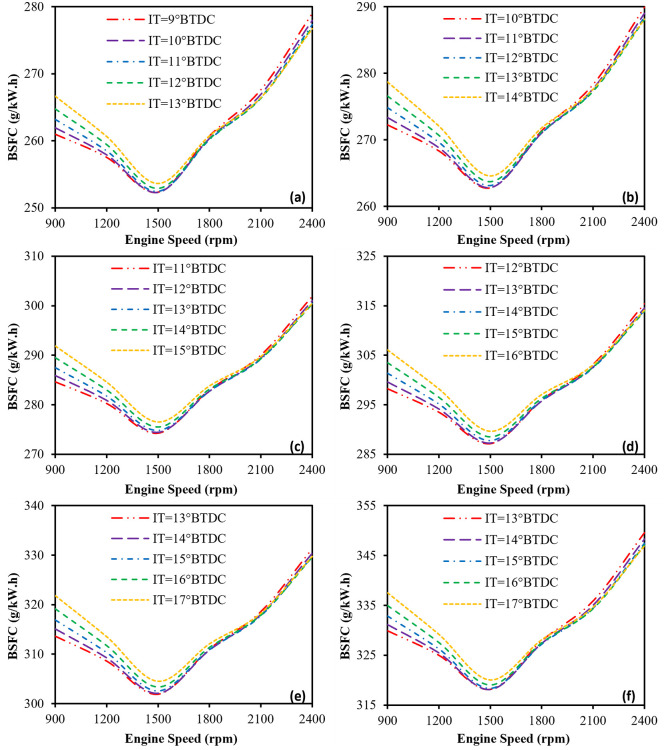
Effect of ignition timing on BSFC at 85% engine load for various blending ratios at CD100 of (a) D100, (b) D90M10, (c) D80M20, (d) D70M30, (e) D60M40, and (f) D50M50.

**Fig 12:**
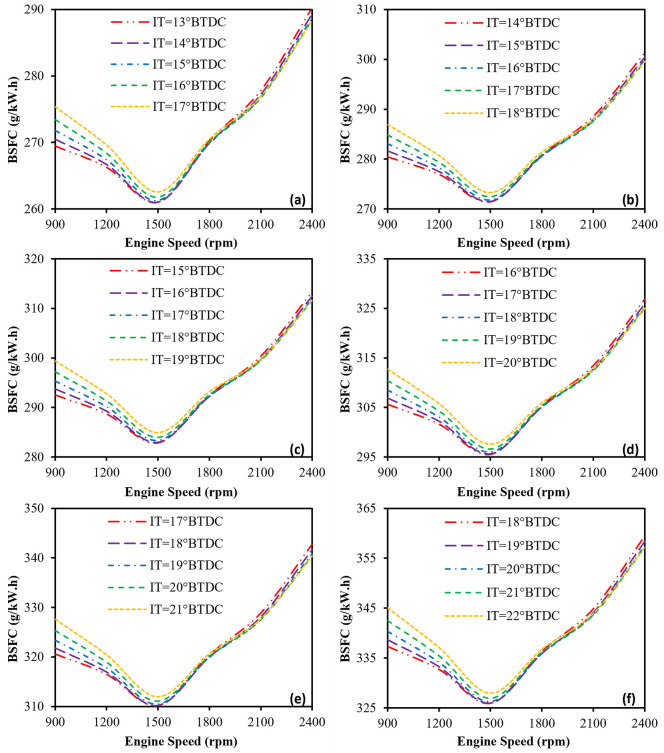
Effect of ignition timing on BSFC at 85% engine load for various blending ratios at CD120 of (a) D100, (b) D90M10, (c) D80M20, (d) D70M30, (e) D60M40, and (f) D50M50.

Figs 9–12 illustrate the influence of IT on brake specific fuel consumption (BSFC) under different blending ratios of (a) D100, (b) D90M10, (c) D80M20, (d) D70M30, (e) D60M40, and (f) D50M50 at 85% load. In the graphs, ignition timing is represented using symbols such as IT = 1^o^BTDC, which denotes combustion taking place 1 degree before top dead center. As IT is advanced, the BSFC increases at low engine speeds but decreases at high engine speeds. The main aspect affecting the BSFC is the inability to convert chemical energy into mechanical energy. Cylinder pressure, fuel mixing, net heat release rate, and volumetric efficiency are highly correlated with it [68,69]. Notably, BSFC decreases as the load increases. Although a higher load requires a greater amount of injected fuel, the rate of power increase exceeds the rate of fuel consumption [70]. The rise in cylinder temperature at higher engine loads reduces the significance of methanol’s high latent heat of vaporization. Meanwhile, the oxygen present in methanol improves combustion efficiency, and together these factors lower BSFC. The decreased density and viscosity of the diesel–methanol mixture enhance fuel atomization and flow characteristics, thereby improving fuel consumption under high-load conditions [71]. Moreover, increasing the methanol BR in the fuel mixture leads to higher BSFC. Because methanol’s calorific value is less than half that of diesel—23.8 MJ/kg compared with 44.5 MJ/kg—it provides significantly less energy per unit mass. Lower cetane rating extends ignition delay, causing more combustion to occur in the expansion stroke, thereby reducing useful work and increasing specific fuel consumption. Therefore, when methanol is blended, a larger fuel quantity is needed while the corresponding increase in power remains limited, resulting in higher BSFC. The BSFC data for the different fuel blends—ranging from D100 to D50M50—at both 70% and 50% engine load are provided in S2 File.

### The predictive model for minimum brake-specific fuel consumption at 50, 70, and 85% engine loads

**Fig 13:**
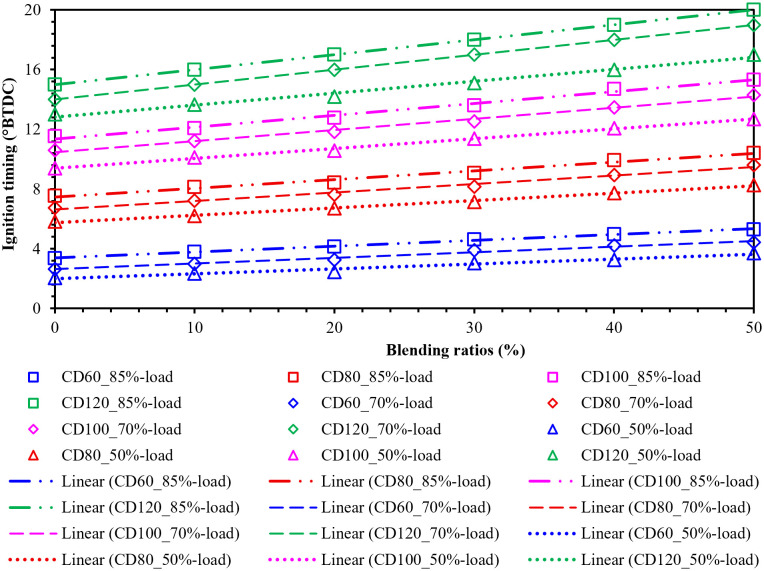
The predictive model of the optimum ignition timing for minimal BSFC at 85, 70, and 50% engine loads.

Internal combustion engines generally operate efficiently within the speed range between maximum torque and maximum power. Therefore, this study determined the optimal IT by identifying the minimum BSFC across the 1500–2400 rpm range. Fig 13 shows the optimum ITs for minimal BSFC at different loads, CDs, and BRs. The greater the methanol content in the blend, the later the optimal IT. The decrease in load causes an IT delay. As the methanol BR in the fuel mixture increases from 0 to 50%, the ideal IT shifts by approximately 1^o^CA to 5^o^CA. The ideal IT also changes by about 1^o^CA to 3^o^CA as the load decreases from 85 to 50%. Additionally, when the CD increases from 60^o^CA to 120^o^CA at 85, 70, and 50% load, respectively, the ideal IT varies by roughly 11^o^CA to 15^o^CA. Based on these results, the optimal IT for achieving minimum BSFC is most strongly influenced by CD, followed by the methanol BR, and lastly by load. The relative impact of these parameters mirrors the trends previously identified for the IT that yields the highest power.

Furthermore, Fig 13 depicts the predictive models developed to determine the optimal IT for minimizing BSFC, formulated as functions of fuel BR and engine load. The graph shows a linear relationship among the three values: load, CD, and BR. Moreover, a predictive framework was established to estimate the IT that yields minimum BSFC based on the BR under various CDs and load conditions, with the corresponding coefficients of determination (R²) reported in Table 7.

**Table 7:** Predictive models for optimal ignition timing for minimizing BSFC in relation to the blending ratio.

CD (^o^CA)	Predictive optimal ignition timing model		
	85% engine load	70% engine load	50% engine load
60	IT = 0.0394BR + 3.3757R² = 0.9978	IT = 0.0376BR + 2.6276R² = 0.9813	IT = 0.033BR + 1.9824R² = 0.9737
80	IT = 0.0582BR + 7.4676R² = 0.9845	IT = 0.0569BR + 6.6281R² = 0.9853	IT = 0.049BR + 5.7543R² = 0.9945
100	IT = 0.0792BR + 11.352R² = 0.9895	IT = 0.0743BR + 10.466R² = 0.9925	IT = 0.0661BR + 9.3838R² = 0.9963
120	IT = 0.1BR + 15R² = 1	IT = 0.1BR + 14R² = 1	IT = 0.0799BR + 12.829R² = 0.9879

## Conclusions

In this study, the effects of different ignition timings (IT), combustion durations (CD), and loads on the performance of a single-cylinder diesel engine fueled with diesel-methanol were investigated through simulation using the AVL Boost software. The engine simulation model in the software is validated, with simulated and experimental values differing by less than 5%. An assessment is conducted to determine the IT that yields both peak torque and the lowest brake specific fuel consumption (BSFC) for methanol–diesel blends with the volume proportion of methanol from 0–50% corresponding to D100, D90M10, D80M20, D70M30, D60M40, and D50M50, considering engine loads of 50, 70, and 85% and CDs spanning 60–120^o^CA. Finally, predictive models based on the optimal ITs are developed. From the study, the following summaries are made:

- Blending ratios (BR), CDs, and loads all affect power output. Power increases as the BRs and loads increase, whereas it decreases when the CDs increase. Along with this, BSFC increases as the BR increases and decreases as the load increases.
- The optimal ITs for maximum power and minimum BSFC are influenced by CDs, BRs, and loads. A higher methanol proportion in the blend leads to a greater retardation of the optimal IT. The maximum deviation in ideal IT between D100 and D50M50 reaches about 5^o^CA for both peak power output and the lowest BSFC conditions. In terms of engine loading, higher loads require more advanced IT. The largest shift in ideal IT when comparing 85 and 50% load amounts to approximately 4^o^CA for the greatest power and around 3^o^CA for minimum BSFC. Finally, regarding CDs, a higher CD leads to an earlier optimal IT. The maximum deviation in optimal IT between CD 60^o^CA and CD 120^o^CA reaches 15^o^CA for both peak power output and the lowest BSFC.
- Predictive models for the optimal ITs corresponding to maximum power and minimum BSFC have been established. The formulations based on CDs, BRs, and engine loads indicate that the relationship between optimal ITs and CDs is linear across all investigated BRs and load conditions. These functions show comparable trends and clearly illustrate the relative effects of CD, BR, and engine load on the optimal IT required to attain peak power output and the lowest BSFC. CD exerts the most potent effect, followed by BRs, while engine loads show the least negligible impact.

To obtain the optimal IT from the proposed prediction equations, two key inputs—engine load and the diesel–methanol BR—must first be determined experimentally. With these predictive models, manufacturers and researchers can estimate the optimal IT according to specific objectives, such as maximizing power output or minimizing BSFC to improve fuel economy. After the optimal IT is identified, the corresponding optimal injection timing can be further derived using the predicted ignition delay, thereby reducing the time, cost, and resources required for extensive experimental campaigns. Importantly, establishing the optimal-IT relationship for diesel–methanol blends is also valuable for determining and calibrating the start-of-combustion phasing in combustion simulations, improving the reliability of numerical studies. It is worth noting that these outcomes provide practical reference data for optimizing injection timing in both experimental investigations and real-world operation of internal combustion engines fueled by diesel–methanol dual-fuel mixtures.

### NOMENCLATURE

**Table:** 

Variables and functions			
*A*_*i*_	surface area (m^2^)	*Q*	the total thermal input from fuel (J)
*a*	vibe parameter	*Q*_*w*_	heat loss through the wall (J)
*BR*	blending ratio	*Q*_*Wb*_	Heat loss from burned zone to wall (J)
*C*_*l*_ *,C*_*2*_	gas velocity coefficient	*Q*_*Wu*_	Heat loss from unburned zone to wall (J)
*C*_*m*_	mean piston velocity (m.s^-1^)	*Q*_*F*_	energy of fuel (J)
*C*_*u*_	circumferential velocity (m.s^-1^)	*T*_*c*_	cylinder temperature (K)
*D*	cylinder bore (m)	*T*_*c,1*_	temperature of cylinder at the intake valve closes (K)
*D100*	pure diesel	*T*_*wi*_	temperature of the wall (K)
*D90M10*	90% diesel blended with 10% methanol	*V*_*D*_	displacement in each cylinder (m^3^)
*D80M20*	80% diesel blended with 20% methanol	*Tor*_*simul*_	simulation brake torque (Nm)
*D70M30*	70% diesel blended with 30% methanol	*V*	volume in the cylinder (m^3^)
*D60M40*	60% diesel blended with 40% methanol	*V*_*b*_	volume of burned zone (m^3^)
*D50M50*	50% diesel blended with 50% methanol	*V*_*u*_	volume of unburned zone (m^3^)
*f*	proportion of the cylinder charge’s evaporation heat	*V*_*c,1*_	volume of the cylinder at the intake valve closes (m^3^)
*h*_*BB*_	specific enthalpy of blow-by (J/kg)	*u*	specific internal energy (J/kg)
*h*_*BB,b*_	specific enthalpy of blow-by gases (burned zone) (J/kg)	*u*_*b*_	specific internal energy of burned gas (J/kg)
*h*_*BB,u*_	specific enthalpy of blow-by gases (unburned zone) (J/kg)	*u*_*u*_	specific internal energy of unburned gas (J/kg)
*h*_*u*_	specific enthalpy of the unburned mixture (J/kg)	*x*	independent parameter
*h*_*i*_	in-flowing mass’s specific enthalpy (J/kg)	***Greek Symbols***	
*h*_*e*_	mass’s specific enthalpy when it exits the cylinder (J/kg)	*α*_*w*_	temperature-transfer efficiency
*m*_*b*_	mass of burned gas (kg)	*α*	angle of crankshaft (deg)
*m*_*BB*_	blow-by mass (kg)	*α*_*o*_	start of combustion
*m*_*BB,b*_	blow-by mass from unburned zone (kg)	Δ*α*_*c*_	combustion duration
*m*_*BB,u*_	blow-by mass from unburned zone (kg)	***Abbreviations***	
*m*_*c*_	mass in the cylinder (kg)	BMEP	brake mean effective pressure
*m*_*ev*_	fuel evaporation (kg)	BMEP_simul_	simulation brake mean effective pressure
*m*_*e*_	mass component ejected from the cylinder (kg)	BTDC	before top dead center
*m*_*i*_	mass component entering the cylinder (kg)	BSFC	brake-specific fuel consumption
*m*_*u*_	mass of unburned gas (kg)	CA	crank angle
*m*	the shape parameter	CD	combustion duration
*N*	engine speed (rpm)	FMEP	friction mean effective pressure
*P*_*simul*_	simulation power (kW)	FMEP_simul_	simulation friction mean effective pressure
*p*_*c*_	cylinder pressure (bar)	HRR	heat release rate
*p*_*c,0*_	cylinder pressure of motored engine (bar)	IMEP	indicated mean effective pressure
*p*_*c,1*_	pressure cylinder at the intake valve closes (bar)	IMEP_simul_	simulation indicated mean effective pressure
*q*_*ev*_	heat of the fuel’s evaporation (J)	IT	ignition timing
*R* ^*2*^	correlation between two variables		

## Supporting information

S1 File Descriptions of experimental apparatus, uncertainty, and margin of error. (DOCX)

S2 File Brake specific fuel consumption of methanol–diesel blends at 70% and 50% engine loads. (DOCX)
