# Supplementary material for: Determine the optimal ignition timing function based on combustion duration, load and fuel blending ratio of an engine powered with diesel-methanol blends
Source: PLoS One. 2026 Jun 22;21(6):e0351949. doi: 10.1371/journal.pone.0351949 (PMC13286148; doi:10.1371/journal.pone.0351949)
Supplement: S1 File — (DOCX) [file pone.0351949.s001.docx]

**Descriptions of experimental apparatus, uncertainty, and margin of error**

All experimental data used in this study were referenced in Ref. [52]. The experiments were conducted in various engine fuel rack settings, and all engine operating condition data and performance were harvested. Then, the engine performance of a certain load (50, 70, and 85% loads) can be extracted from the obtained data. The experimental apparatus is presented in Fig S1, with Vikyno RV125-2 engine and generator (1), computer (2), controller (3), heat resistance loader (4), load cell (5), fuel tank (6), temperature data logger (7), emergency stop device (8), and air cleaner and flow meter sensor (9). Torque measurements were carried out with a BCA-15L load cell (range: 75 kgf, error: ±0.06%), while engine rotational speed was determined from the generator output frequency. The intake air mass flow rate was measured with a Sensyflow FMT700-P flow meter (range: 720 kg/h, error: ±1%), while the fuel mass flow rate was recorded using an electronic balance weighing scale of Jadever JWE-15K (range: 15,000 g, resolution: 0.5 g). The uncertainty of measured and calculated parameters was computed using Eq. (S1-1) [S1] and summarized in Table S1-I.


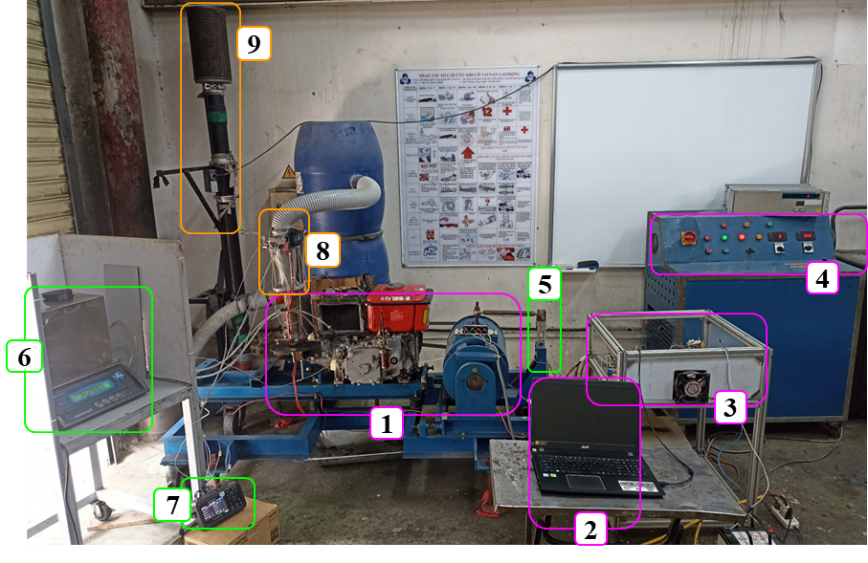


Fig S1. The experiment set-up [59].

$w_{X}=\left\{\left[\left(\partial X/\partial x_{1}\right)\cdot w_{1}\right]^{2}+\left[\left(\partial X/\partial x_{2}\right)\cdot w_{2}\right]^{2}+...+\left[\left(\partial X/\partial x_{n}\right)\cdot w_{n}\right]^{2}\right\}^{0.5}$ (S1-1)

where *X* is a calculated parameter, *x_1_, x_2_,* and *x_n_* are the independent parameters, *w* is the uncertainty.

Table S1-I. Uncertainties of the measured and calculated parameters

| Parameters | Uncertainty (%) |
| --- | --- |
| Torque | ± 0.06 |
| Speed | ± 0.11 |
| Air flow rate | ± 1 |
| Fuel mass flow rate | ± 2.5 |
| Power | ± 0.13 |

The margin of errors was calculated by using Eqs. (S1-2) and (S1-3) with the confidence intervals of 95 % and the number of samples of 3 [S2]:

$MOE=4.303\frac{S}{\sqrt{n}}$ (S1-2)

$S=\sqrt{\frac{(v_{i}-\bar{v})^{2}}{n-1}}$ (S1-3)

where *MOE* is the margin of error, *S* is the standard deviation, *v_i_* and $\bar{\text{v}}$ are the observed and average values, respectively, *n* is the number of samples.

**References**

52. Hong TD, Hoang BTT, Ho TQM, Truong PT, Pham MQ. An experimental investigation on the performance of a single cylinder diesel engine at various partial loads. Transportation Engineering. 2024;18: 100288. doi:10.1016/j.treng.2024.100288

S1 Taylor JR. An Introduction to Error Analysis: The Study of Uncertainties in Physical Measurements. 2nd ed. Sausalito: University Science Books; 1997.

S2 Moore DS, McCabe GP, Craig BA. Introduction to the Practice of Statistics. 9th ed. New York: W. H. Freeman and Company; 2016.
