# Supplementary material for: Determine the optimal ignition timing function based on combustion duration, load and fuel blending ratio of an engine powered with diesel-methanol blends
Source: PLoS One. 2026 Jun 22;21(6):e0351949. doi: 10.1371/journal.pone.0351949 (PMC13286148; doi:10.1371/journal.pone.0351949)
Supplement: S2 File — (DOCX) [file pone.0351949.s002.docx]

**Brake specific fuel consumption (BSFC) of methanol–diesel blends at 70% and 50% engine loads under combustion durations of 60°CA, 80°CA, 100°CA, and 120°CA.**

In this study, the optimal ignition timings (ITs) were investigated under various engine loads and combustion durations for different blending ratios (BRs). Figs S1 to S8 illustrate the brake specific fuel consumption (BSFC) of different BRs at various ITs under different combustion durations at 50% and 70% engine loads.

**Fig S1. Effect of ignition timing on BSFC at 70% engine load for various blending ratios at a combustion duration of 60°CA: (a) D100, (b) D90M10, (c) D80M20, (d) D70M30, (e) D60M40, and (f) D50M50.**

**Fig S2. Effect of ignition timing on BSFC at 70% engine load for various blending ratios at a combustion duration of 80°CA: (a) D100, (b) D90M10, (c) D80M20, (d) D70M30, (e) D60M40, and (f) D50M50.**

**Fig S3. Effect of ignition timing on BSFC at 70% engine load for various blending ratios at a combustion duration of 100°CA: (a) D100, (b) D90M10, (c) D80M20, (d) D70M30, (e) D60M40, and (f) D50M50.**

**Fig S4.** **Effect of ignition timing on BSFC at 70% engine load for various blending ratios at a combustion duration of 120°CA: (a) D100, (b) D90M10, (c) D80M20, (d) D70M30, (e) D60M40, and (f) D50M50.**

**Fig S5. Effect of ignition timing on BSFC at 50% engine load for various blending ratios at a combustion duration of 60°CA: (a) D100, (b) D90M10, (c) D80M20, (d) D70M30, (e) D60M40, and (f) D50M50.**

**Fig S6.** **Effect of ignition timing on BSFC at 50% engine load for various blending ratios at a combustion duration of 80°CA: (a) D100, (b) D90M10, (c) D80M20, (d) D70M30, (e) D60M40, and (f) D50M50.**

**Fig S7.** **Effect of ignition timing on BSFC at 50% engine load for various blending ratios at a combustion duration of 100°CA: (a) D100, (b) D90M10, (c) D80M20, (d) D70M30, (e) D60M40, and (f) D50M50.**

**Fig S8.** **Effect of ignition timing on BSFC at 50% engine load for various blending ratios at a combustion duration of 120°CA: (a) D100, (b) D90M10, (c) D80M20, (d) D70M30, (e) D60M40, and (f) D50M50.**
